# Improved survival of hospitalized patients with cardiac arrest due to coronary heart disease after implementation of post-cardiac arrest care: A population-based study

**DOI:** 10.1097/MD.0000000000012382

**Published:** 2018-09-14

**Authors:** Yu Lin, Shih-Hung Tsai, Chen-Shu Yang, Chun-Hsien Wu, Chih-Han Huang, Fu-Huang Lin, Chih-Hung Ku, Chi-Hsiang Chung, Wu-Chien Chien, Chung-Yu Lai, Chi-Ming Chu

**Affiliations:** a Graduate Institute of Life Sciences; b Department of Nursing, University of Kang Ning; c Department of Emergency Medicine; d Physical Examination Center, Kaohsiung Armed Forces General Hospital Gangshan Branch; e Division of Cardiology; f Division of Cardiovascular Surgery; g School of Public Health; h Department of Health Industry Management, Kainan University, Taoyuan City; i Department of Medical Research, Tri-Service General Hospital, National Defense Medical Center, Taipei City; j Department of Healthcare Administration and Medical Informatics College of Health Sciences, Kaohsiung Medical University; k Department of Medical Research, Kaohsiung Medical University Hospital, Kaohsiung City, Taiwan.

**Keywords:** coronary heart disease, International Classification of Disease Clinical Modification, 9th revision codes, National Health Insurance Research Database, post-cardiac arrest care

## Abstract

Post-cardiac arrest care was implemented in 2010 and has been shown to improve the survival of patients with coronary heart disease (CHD). However, the findings varied for different survival conditions.

We conducted a retrospective longitudinal study of records from 2007 to 2013 in the National Health Insurance Research Database. We evaluated the differences in short-term (2-day and 7-day) and long-term (30-day and survival to discharge) survival after the implementation of post-cardiac arrest care and among age subgroups. We reviewed inpatient datasets in accordance with the International Classification of Disease Clinical Modification, 9th revision codes (ICD-9-CM). Eligible participants were identified as those with simultaneous diagnoses of cardiac arrest (ICD-9-CM codes: 427.41 or 427.5) and CHD (ICD-9-CM codes: 410–414). Multiple logistic regression was applied to establish the relationship between calendar year and survival outcomes.

The odds of 2-day survival from 2011 to 2013 were higher than those from 2007 to 2010 (adjusted odds ratio [aOR]: 1.15; 95% confidence interval [CI]: 1.03–1.29). Similarly, the odds of 7-day survival from 2011 to 2013 were higher than those from 2007 to 2010 (aOR: 1.11; 95% CI: 1.01–1.22). Improvements in the odds of 2-day and 7-day survival were discovered only in patients <65 years old. Our data reinforce that short-term survival improved after implementation of post-cardiac arrest care. However, older age seemed to nullify the influence of post-cardiac arrest care on survival.

## 1 Introduction

The introduction of a standard resuscitation protocol has increased the survival rate of cardiac arrest patients. However, most cardiac arrest patients who have achieved a return of spontaneous circulation (ROSC) experience post-resuscitation syndrome (e.g., neurological failure) and still have a high in-hospital mortality rate.^[1,2]^ In the 2010 American Heart Association Guidelines for Cardiopulmonary Resuscitation and Emergency Cardiovascular Care, post-cardiac arrest care was added as the 5th link to the adult chain of survival. Consequently, a tight connection among the 5 links of the survival chain is crucial for improving the prognosis of out-of-hospital cardiac arrest events.^[3,4]^ Post-cardiac arrest care has also been recognized to improve the survival of in-hospital cardiac arrest patients.^[5]^

For several decades, coronary heart disease (CHD) has remained the leading cause of mortality, and the burden of this disease is immensely high in the healthcare systems of developed countries. CHD is common in patients who experience sudden cardiac arrest.^[6,7]^ The main etiology of post-cardiac arrest survivors has been identified as acute CHD, such as myocardial infarction.^[5]^ Historically, emerging research has suggested that optimized post-cardiac arrest care, including rapid assessment and intervention for cardiac arrest caused by CHD, can improve the chances of survival and recovery for victims.^[8,9]^

However, certain studies have indicated an increased rate of ROSC without an improvement in long-term survival.^[10]^ To clarify the effect of post-cardiac arrest care on various prognoses, more nationwide population-based studies are necessary. In this study, we evaluated whether survival differences exist among hospitalized cardiac arrest cases due to CHD before and after the implementation of post-cardiac arrest care. Furthermore, we determined the impact of age on survival during hospitalization.

## 2 Methods

### 2.1 Data resource and study design

In 1995, a single-payer National Health Insurance program was launched in Taiwan by legislation. As of 2014, this worldwide-renowned health insurance system covered up to 99.9% of Taiwan's population.^[11]^ The Digital National Health Insurance Research Database (NHIRD) was derived from this system by the National Health Insurance Administration and released for analysis by the National Health Research Institutes. The scrambled NHIRD contains inpatient and outpatient medical records that cannot be linked to the identities of individual patients or facilities to protect privacy. We conducted this 7-year retrospective longitudinal study using the inpatient NHIRD covering the time period of January 1, 2007 through December 31, 2013. This study was assessed as low risk and approved by the Institutional Review Board of Tri-Service General Hospital in Taipei City, Taiwan.

### 2.2 Eligible participants and exclusive criteria

We reviewed the inpatient expenditures in the admissions dataset, which documented medical diagnoses and treatments in accordance with the International Classification of Disease Clinical Modification, 9th revision codes (ICD-9-CM). Eligible participants were identified as those with simultaneous diagnoses of cardiac arrest (ICD-9-CM codes: 427.41 or 427.5)^[12]^ and CHD (ICD-9-CM codes: 410–414).^[13]^ We also searched the procedural codes of cardiac catheterization, coronary arteriography (37.21–37.23 and 88.52–88.57), and percutaneous transluminal coronary angioplasty (PTCA) (00.66, 17.55, 36.01, 36.02, and 36.05–36.07).^[14]^

In total, 8077 cases were recruited from the NHIRD; 32 patients were excluded from the study because they corresponded to one of the following criteria: suffered from cardiac arrest before the 2007 calendar year (n = 11); <18 years of age (n = 13); recording of an indefinite survival outcome (n = 4); or lost to follow-up (n = 4). Finally, 8045 hospitalized patients with cardiac arrest due to CHD were included in the analysis.

### 2.3 Outcome variables

To determine the influence of the implementation of post-cardiac arrest care on survival, different types of outcome variables were calculated in accordance with the length of hospital stay. Short-term and long-term survival rates were measured for comparison between different subgroups in the calendar year depending on the time at which post-cardiac arrest care was delivered (2007–2010 and 2011–2013). Short-term survival included 2-day survival or hospital discharge (2-day survival) and 7-day survival or hospital discharge (7-day survival); long-term survival included 30-day survival or discharge (30-day survival) and survival to discharge.

### 2.4 Statistical analysis

We calculated the percentage of each variable for descriptive statistics. The chi-square test was used to examine the differences in proportions between calendar year subcategories (2007–2010 and 2011–2013). We included the following variables: age (stratified <45, 45–64, 65–79, and ≥80 years of age), sex, Charlson Comorbidity Index (CCI) (stratified 0, 1, and ≥2),^[15]^ hospital level (medical center, regional/local hospital), cardiac rhythm on admission (ventricle fibrillation [VF], non-VF), coronary catheterization (no, yes), PTCA (no, yes), length of stay (stratified 1, 2–6, 7–29, and ≥30 days), and survival condition (2-day, 7-day, 30-day survival, survival to discharge).

Variables with two-tailed *P* values <.10 according to the univariate analysis were enrolled in the multivariate analysis. Multiple logistic regression with the ENTER model was applied to establish the relationship between calendar year and survival outcome. The statistical analysis in this study was performed using SPSS 22 (IBM, Armonk, NY).

## 3 Results

From 2007 to 2013, a total of 8045 cardiac arrest patients survived upon hospitalization due to CHD. A total of 32.9% of patients were 45 to 64 years of age, and 36.3% were 65 to 79 years of age. There were 66.8% male and 33.2% female patients. The proportions of CCI scores of 1 and ≥2 were 31.3% and 15.0%, respectively, in comorbidity conditions. In addition, 39.5% of subjects were treated at a high-capacity medical center. There were statistically significant differences in the variables mentioned above among the calendar year subgroups (2007–2010 vs 2011–2013), as shown in Table 1.

**Table 1:**
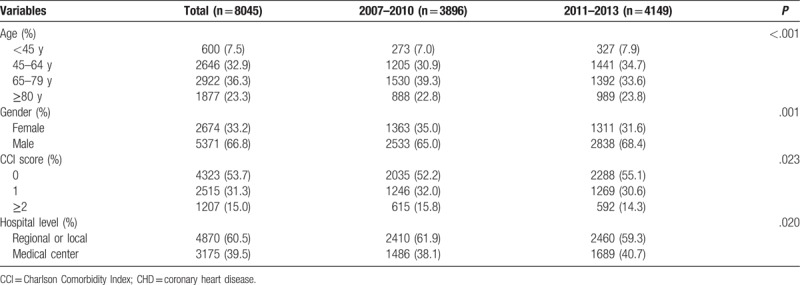
Characteristics of hospitalized patients with cardiac arrest due to CHD.

**Figure 1:**
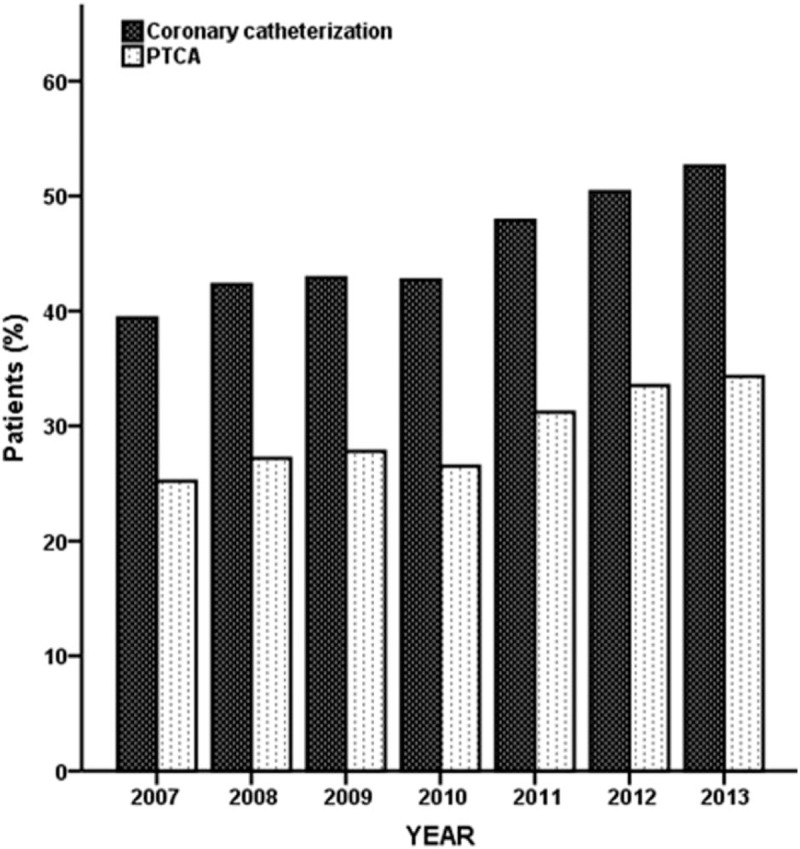
Percentage of hospitalized patients with cardiac arrest due to CHD undergoing coronary catheterization and PTCA from 2007 to 2013. CHD = coronary heart disease and PTCA = percutaneous transluminal coronary angioplasty.

The data in Table 2 show that 41.6% of patients had VF of cardiac rhythm on admission. The percentages of patients who underwent coronary catheterization and PTCA were 46.3% and 30.0%, respectively. In addition, we observed an increasing year-to-year trend (*P* for trend: <.001), as depicted in Fig. 1. Furthermore, 23.7% of patients had a hospital stay of 1 day, and 27.8%, 35.8%, and 12.8% of patients had a hospital stay of 2 to 6 days, 7 to 29 days, and ≥30 days, respectively. The distributions of the variables of cardiac rhythm on admission, coronary catheterization, PTCA, and length of stay differed between the 2 patient subgroups based on calendar year.

**Table 2:**
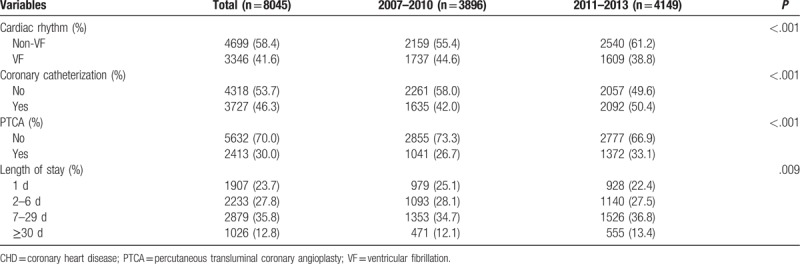
Clinical descriptions of hospitalized patients with cardiac arrest due to CHD.

The prognosis of post-cardiac arrest patients is illustrated in Table 3. The percentages of 2-day, 7-day, and 30-day survival among the subjects were 78.1%, 62.5%, and 49.5%, respectively. For long-term survival, 44.2% of the study population survived to hospital discharge. However, according to the univariate analysis, the survival rates did not significantly differ between the 2007 to 2010 and 2011 to 2013 calendar year patient subgroups.

**Table 3:**
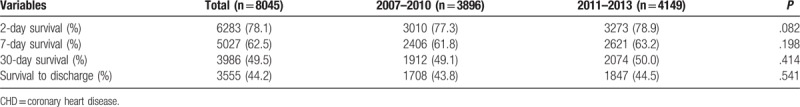
Survival outcomes of hospitalized patients with cardiac arrest due to CHD.

After adjustments for age, sex, CCI, and hospital level, the odds of 2-day survival from 2011 to 2013 were higher than those from 2007 to 2010 (adjusted odds ratio [OR]: 1.15; confidence interval [CI]: 1.03–1.29; *P* = .011). Similarly, the odds of 7-day survival from 2011 to 2013 were higher than those from 2007 to 2010, as shown in Table 4. Stratified by age group, as shown in Table 5, the odds of 2-day and 7-day survival were improved from 2011 to 2013 compared with 2007 to 2010 for only patients <65 years.

**Table 4:**
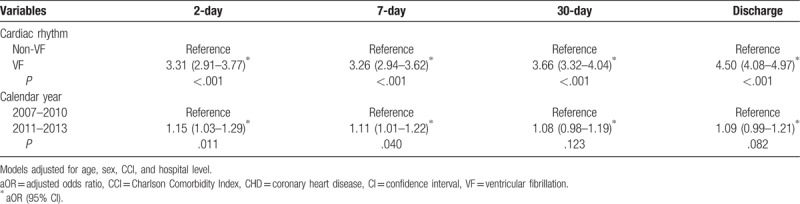
Adjusted odds ratios of the time effect on survival conditions among hospitalized patients with cardiac arrest due to CHD.

**Table 5:**
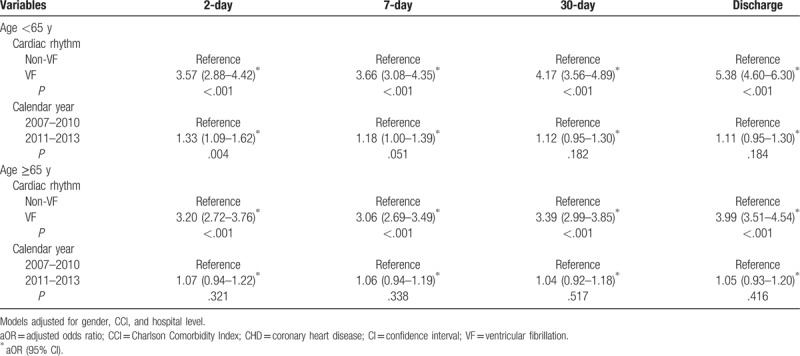
Adjusted odds ratios of the time effect on different survival conditions among hospitalized patients with cardiac arrest due to CHD stratified by age.

## 4 Discussion

In this observational population-based study, we present the survival patterns of patients post-cardiac arrest due to CHD between 2007 and 2013 using the NHIRD. There was a significant temporal increase in the proportion of hospitalized patients with cardiac arrest due to CHD undergoing coronary catheterization and PTCA over the study period. After controlling for age, sex, CCI, and hospital level, the calendar year interval during which the arrest event occurred remained a positive indicator of 2-day and 7-day survival. VF cardiac rhythm on admission has been widely considered to be the strongest variable affecting outcome. However, these results indicated that there was a survival improvement for only patients <65 years of age during the 2011 to 2013 time period.

Shin et al^[16]^ described that there was a trend of increased odds of survival from cardiac arrest over time. Dudas et al^[13]^ found that the percentage of patients with CHD who died in the hospital or within 28 days of hospitalization among out-of-hospital deaths decreased over time in Germany. A study conducted by Fugate et al^[14]^ showed that post-cardiac arrest morality has significantly declined from 69.6% to 57.8% in the United States. Girotra et al^[17]^ also implied that rates of post-resuscitation survival to hospital discharge substantially improved in northern America over the study period. In summary, a number of studies have suggested a temporally improving trend in the rate of survival. Since 2010, a fifth link has been added to the chain of survival to emphasize the importance of post-cardiac arrest care, such as early coronary catheterization and admission to the intensive care unit. Therefore, our study assessed different types of survival rates among hospitalized patients with cardiac arrest due to CHD. Compared with preceding studies, our results also reflect an improvement in short-term survival. However, published statistical data have consistently indicated that many interventions to treat cardiac arrest patients increase the rate of short-term survival (e.g., ROSC) but not long-term survival. This point needs to be further addressed in the future by more large-scale studies worldwide.

Cardiac rhythm is well known as the most common parameter correlated with prognosis. Regarding cardiac rhythm, numerous prior studies have reported that patients coded with VF rhythm had a greater chance of survival after resuscitation.^[18–20]^ Moreover, cardiac arrest victims exhibit a higher VF/VT rhythm ratio due to cardiac etiologies.^[7,21]^ Approximately 40% of cardiac arrest patients had VF rhythm in our study, which might be due to our selection of hospitalized patients with cardiac arrest due to CHD, whom tend to present VF rhythm on admission to the emergency room and post discharge. Coronary catheterization or PTCA treatment has been verified to improve the survival outcomes of cardiac arrest patients.^[5,22,23]^ There was a significant temporal increase in the percentage of coronary catheterization or PTCA intervention among our study subjects relative to those in a former report.^[14]^ In other words, coronary catheterization and PTCA were highly correlated with the year during which cardiac arrest occurred. In the analysis, we used the calendar year subgroup as a variable in the multivariate regression model and then highlighted the effect of time on survival after modification of the survival chain.

The goal of this study was to introduce changes in short-term and long-term survival after the implementation of post-cardiac arrest care. From reviewing data in the literature, the possibility of a favorable survival outcome is dramatically reduced among cardiac arrest patients ≥65 years of age.^[13,14,24]^ We further performed a stratified statistical analysis to explore how age modified the effect of time on independent outcomes. Our findings imply that there is an increased opportunity for short-term survival only among young subjects <65 years of age. In the future, we will extend this study to clarify this disparity among the elderly population.

## 5 Limitations

There were several inherent limitations in this study. First, due to database restrictions, we did not have information pertaining to existing clinical symptoms, such as cerebral performance scores, post-cardiac arrest syndrome, and reperfusion injury,^[25]^ which may contribute to a residual confounding effect. Second, we recruited only hospitalized patients with cardiac arrest due to the characteristics of the database. Although information pertaining to some of the subjects was missing, our study confirms the significance of post-cardiac arrest care on CHD. In addition, varying definitions of short-term and long-term survival have been utilized across studies. In this study, we used the length of stay in the hospital to calculate several survival outcomes. However, for example, 2-day survival included patients who survived <48 hours during hospitalization, as the exact survival time is not recorded. To reduce the impact of nondifferential misclassification on the estimation of short-term survival, we additionally calculated 7-day survival to represent the influence of post-cardiac arrest care on another short-term variable. Finally, when patients survived to hospital discharge, we could not continue to follow up on their outcome, which may have limited our ability to evaluate the contribution of post-cardiac arrest care.

## 6 Conclusions

We show that short-term survival was improved after the implementation of post-cardiac arrest care in patients with CHD in Taiwan. Subjects with VF rhythm upon admission had a better prognosis in the phase with post-cardiac arrest care than in that without post-cardiac arrest care. Through stratification, our findings revealed that older age seemed to nullify the influence of post-cardiac arrest care on survival.

## Acknowledgments

This study was based in part on data from the NHIRD provided by the NHIA and Ministry of Health and Welfare and managed by the NHRI. The interpretation and conclusions contained herein do not represent the views of the NHIA or NHRI. The authors thank Pai Lu for the consultation on the statistical analysis.

## Author contributions

**Conceptualization:** Shih-Hung Tsai, Chen-Shu Yang, Chung-Yu Lai.

**Formal analysis:** Yu Lin, Fu-Huang Lin, Chi-Hsiang Chung, Chung-Yu Lai, Chi-Ming Chu.

**Methodology:** Shih-Hung Tsai, Chen-Shu Yang, Chun-Hsien Wu, Chih-Han Huang.

**Project administration:** Fu-Huang Lin.

**Resources:** Fu-Huang Lin, Wu-Chien Chien.

**Software:** Chi-Hsiang Chung.

**Writing – original draft:** Yu Lin, Chung-Yu Lai.

**Writing – review & editing:** Yu Lin, Shih-Hung Tsai, Chen-Shu Yang, Chun-Hsien Wu, Chih-Han Huang, Chih-Hung Ku, Wu-Chien Chien, Chung-Yu Lai, Chi-Ming Chu.
